# Supplementary material for: The Systems Biology Research Tool: evolvable open-source software
Source: BMC Syst Biol. 2008 Jun 29;2:55. doi: 10.1186/1752-0509-2-55 (PMC2446383; doi:10.1186/1752-0509-2-55)
Supplement: Additional file 1 — SBRT Archive. An archive of the current version of the Systems Biology Research Tool. [file 1752-0509-2-55-S1.zip › sbrt-1.4.0/doc/developers_guide/api/sbrt/shell/text/fba/FbaOptHeaderFormatterV1.html]

FbaOptHeaderFormatterV1


|  |  |  |
| --- | --- | --- |
| **Overview** \| **Package** \| **Class** \| **Use** \| **Tree** \| **Deprecated** \| **Index** \| **Help** | |  |
| **PREV CLASS**   **NEXT CLASS** | **FRAMES**    **NO FRAMES**     **All Classes** |  |
| SUMMARY: NESTED \| FIELD \| CONSTR \| METHOD | DETAIL: FIELD \| CONSTR \| METHOD |  |


---


## sbrt.shell.text.fba Class FbaOptHeaderFormatterV1

```
java.lang.Object
  sbrt.shell.text.fba.FbaOptHeaderFormatterV1
```

**All Implemented Interfaces:**: FbaOptHeaderFormatter, Formatter<java.lang.String>

---

``` public class FbaOptHeaderFormatterV1 extends java.lang.Object implements FbaOptHeaderFormatter ```

This class is a concrete implemenation of `FbaOptHeaderFormatter`.

**Author:**
:   This class was written and documented by Jeremiah Wright while in the
    Wagner lab.

---

| **Field Summary** | |
| --- | --- |
| `static java.lang.String` | `OBJ_VALUE`             The header used to indicate the value of the objective function. |


**Constructor Summary**

`FbaOptHeaderFormatterV1(Fluxome fluxome)`
  
          Constructs a new FBA optimization header formatter.


| **Method Summary** | |
| --- | --- |
| `java.lang.String` | `format(java.lang.String header)`             Returns a formatted string representation of the provided header. |
| `Fluxome` | `getFluxome()`             Returns the fluxome used to verify reaction names. |
| `java.lang.String` | `getObjValueHeader()`             Returns the header used to indicate the value of the objective function. |
| `PipeSetFormat<java.lang.String>` | `getPipeSetFormat()`             Returns the format used for sets of strings. |
| `RxnNameSetFormatV1` | `getRxnNameSetFormat()`             Returns the format used for reaction name sets. |
| `boolean` | `isObjValueHeader(java.lang.String s)`             Indicates if the provided string is the header used to indicate the value of the objective function. |

**Methods inherited from class java.lang.Object**

`clone, equals, finalize, getClass, hashCode, notify, notifyAll, toString, wait, wait, wait`

**Field Detail**

### OBJ\_VALUE

```
public static final java.lang.String OBJ_VALUE
```

:   The header used to indicate the value of the objective function.

    **See Also:**: Constant Field Values


**Constructor Detail**

### FbaOptHeaderFormatterV1

```
public FbaOptHeaderFormatterV1(Fluxome fluxome)
```

:   Constructs a new FBA optimization header formatter.

    **Parameters:**: `fluxome` - the fluxome used to verify reaction names.


**Method Detail**

### getFluxome

```
public Fluxome getFluxome()
```

:   Returns the fluxome used to verify reaction names.

    :   **Specified by:**: `getFluxome` in interface `FbaOptHeaderFormatter`
    :   **Returns:**: the fluxome used to verify reaction names.

---


### getRxnNameSetFormat

```
public RxnNameSetFormatV1 getRxnNameSetFormat()
```

:   Returns the format used for reaction name sets.

    :   **Returns:**: the format used for reaction name sets.

---


### getPipeSetFormat

```
public PipeSetFormat<java.lang.String> getPipeSetFormat()
```

:   Returns the format used for sets of strings.

    :   **Returns:**: the format used for sets of strings.

---


### getObjValueHeader

```
public java.lang.String getObjValueHeader()
```

:   Returns the header used to indicate the value of the objective function.

    :   **Specified by:**: `getObjValueHeader` in interface `FbaOptHeaderFormatter`
    :   **Returns:**: the header used to indicate the value of the objective function.

---


### isObjValueHeader

```
public boolean isObjValueHeader(java.lang.String s)
```

:   Indicates if the provided string is the header used to indicate the value
    of the objective function.

    :   **Specified by:**: `isObjValueHeader` in interface `FbaOptHeaderFormatter`
    :   **Parameters:**: `s` - the string to compare. **Returns:**: `true` if the provided string is equal to the string returned by `getObjValueHeader()`; `false` otherwise.

---


### format

```
public java.lang.String format(java.lang.String header)
```

:   Returns a formatted string representation of the
    provided header.

    :   **Specified by:**: `format` in interface `Formatter<java.lang.String>`
    :   **Parameters:**: `header` - the header to be formatted. **Returns:**: a formatted string representation of the provided header.


---


|  |  |  |
| --- | --- | --- |
| **Overview** \| **Package** \| **Class** \| **Use** \| **Tree** \| **Deprecated** \| **Index** \| **Help** | |  |
| **PREV CLASS**   **NEXT CLASS** | **FRAMES**    **NO FRAMES**     **All Classes** |  |
| SUMMARY: NESTED \| FIELD \| CONSTR \| METHOD | DETAIL: FIELD \| CONSTR \| METHOD |  |


---
